# The Diabetes Manual trial protocol – a cluster randomized controlled trial of a self-management intervention for type 2 diabetes [ISRCTN06315411]

**DOI:** 10.1186/1471-2296-7-45

**Published:** 2006-07-17

**Authors:** Jackie Sturt, Hilary Hearnshaw, Andrew Farmer, Jeremy Dale, Sandra Eldridge

**Affiliations:** 1 Centre for Primary Health Care Studies, Warwick Medical School, University of Warwick, Coventry, CV4 7AL, UK; 2 Department of Primary Health Care, University of Oxford, Old Road Campus, Oxford, OX3 7LF, UK; 3 Institute of Health Sciences Education, Barts and the London Queen Mary Institute of Medicine and Dentistry, Mile End Rd, London, E1 4NS, UK

## Abstract

**Background:**

The Diabetes Manual is a type 2 diabetes self-management programme based upon the clinically effective 'Heart Manual'. The 12 week programme is a complex intervention theoretically underpinned by self-efficacy theory. It is a one to one intervention meeting United Kingdom requirements for structured diabetes-education and is delivered within routine primary care.

**Methods/design:**

In a two-group cluster randomized controlled trial, GP practices are allocated by computer minimisation to an intervention group or a six-month deferred intervention group. We aim to recruit 250 participants from 50 practices across central England. Eligibility criteria are adults able to undertake the programme with type 2 diabetes, not taking insulin, with HbA1c over 8% (first 12 months) and following an agreed protocol change over 7% (months 13 to 18). Following randomisation, intervention nurses receive two-day training and delivered the Diabetes Manual programme to participants. Deferred intervention nurses receive the training following six-month follow-up. Primary outcome is HbA1c with total and HDL cholesterol; blood pressure, body mass index; self-efficacy and quality of life as additional outcomes. Primary analysis is between-group HbA1c differences at 6 months powered to give 80% power to detect a difference in HbA1c of 0.6%. A 12 month cohort analysis will assess maintenance of effect and assess relationship between self-efficacy and outcomes, and a qualitative study is running alongside.

**Discussion:**

This trial incorporates educational and psychological diabetes interventions into a single programme and assesses both clinical and psychosocial outcomes. The trial will increase our understanding of intervention transferability between conditions, those diabetes related health behaviours that are more or less susceptible to change through efficacy enhancing mechanisms and how this impacts on clinical outcomes.

## Background

### International health policy

The past decade has seen an international trend towards providing primary care based diabetes services with patient education and self-management at the forefront. The International Diabetes Foundation (IDF)[1] standards advocate that "implementation of diabetes education is learner-centred, facilitates cognitive learning, behaviour change and self-management". These are challenging goals for health care providers to uphold but nonetheless are being incorporated into national health policies. For example, shared decision making is a standard promoted in Finland [2], the Americas [3] and the Netherlands [4].

In the United Kingdom (UK), health professionals are expected to work with people living with diabetes to develop their confidence, skills and knowledge, engage in shared decision making and to provide theory-based structured education [5-7]. It has been proposed that such education should meet four criteria [8]: (i) have a structured, written curriculum (ii) have trained educators (iii) be quality assured, and (iv) be audited. The majority of people living with type 2 diabetes access education, information and support through primary care [9] which is currently structured, almost exclusively, around individual patient consultations. The potential of practice nurses to support individuals in structured patient self-management education within this existing, and valued, primary care structure needs to be built upon.

### Self-management interventions improve clinical outcomes

Psychological interventions for use in type 2 diabetes have been systematically reviewed and pooled trial results suggest they reduce HbA1c by a clinically significant 1% [10]. The need for research that combines psychological interventions with educational packages to understand the impact of psychological interventions on self-management behaviours has been recognised [11]. Hampson et al [12] reviewed behavioural interventions for adolescents with type 1 diabetes and found that only a minority of studies described interventions that were explicitly based on theories. The theoretically determined interventions, however, generated larger effect sizes than those that were atheoretical. Earlier systematic reviews of patient self-management training in long term conditions [13-15] concluded that collaborative self-management interventions, where people respond to clinical information and goal setting, represent the most effective self-management approaches for improving clinical outcomes. Together, these reviews suggest a need to explore theory-based self-management intervention development, involving biomedical feedback and goal evaluation.

### Self-efficacy theory

Improving an individual's self-efficacy is particularly important in long term disease self-management as it reflects capacity to carry out health related behaviours that are likely to improve outcome[16]. This parameter is based on social learning theory and reflects an individual's level of confidence in their ability to perform particular behaviours. Interventions to increase efficacy based on this theory, include four specific techniques of facilitating personal mastery, vicarious (observing others) learning, identifying distress and providing verbal persuasion. These have been shown to be powerful ways for people to learn new behaviours and activities. Enhancing self-efficacy in long term disease management has been shown to be associated with lower levels of health care consumption and improved psychosocial adjustment [17, 18, 19, 20].

### One to one education

The evidence for the effectiveness of group education is growing, [7,21] but group approaches do not suit everyone [22]. Randomised controlled trials of the Heart Manual [23,24], a home-based one-to-one secondary prevention programme for coronary heart disease, demonstrated findings that were at least equal to group approaches. Dalal & Evans [22] reported that the one to one Heart Manual was 11% more popular with primary care patients than the group alternative. It also had an 87% programme completion rate compared to the 33% completion rate of the group-based programme. The study found those over 60 years, the self-employed and those living in rural locations to particularly prefer an individual approach. Individual approaches and group-based self-management programmes may, therefore, be complementary. Both may be needed to engage the wide constituency of people requiring diabetes self-management education.

### The Diabetes Manual intervention

The Diabetes Manual, combining the three preceding strategies for improving outcomes in a single package, was developed at the University of Warwick in 2003–4, based on the Heart Manual. The development of a programme was prioritised in a needs assessment [25] and its subsequent development was informed by both lay and professional expert panels. Two focus groups of people with type 2 diabetes confirmed that the penultimate draft of the Diabetes Manual had face validity. The Diabetes Manual programme incorporates self-efficacy enhancing text and activities and has been designed to enable patients to gain confidence and skills quickly and progressively in the management of their diabetes[26]. The programme has five components, i) two-day training for nurse to deliver the programme ii) 12 week patient manual iii) relaxation audiotape iv) frequently asked questions audiotape for patient and carers v) telephone support from nurse in weeks 1,5 and 11. The Diabetes Manual is designed to be offered to patients during primary care consultations with GPs and practice nurses and therefore is intended to have high availability to its target population. It is able to meet the UK standards for structured education in diabetes [8]. This paper discusses a trial that is now underway to test the effectiveness of the Diabetes Manual programme.

## Trial objectives

### Primary objective

To determine the efficacy of the Diabetes Manual in type 2 diabetes for improving glycaemic control at 6-months compared with usual care and to determine the persistence of effect at 12 month in the intervention group.

### Secondary objectives

To determine the efficacy of the Diabetes Manual in modifying the following risk factors for cardiovascular disease: total serum cholesterol; HDL cholesterol; blood pressure and BMI. To reduce anxiety and improve quality of life and self-efficacy.

## Methods/design

The Diabetes Manual trial is a two-arm RCT, clustered by GP practice with an intervention arm and a control arm in which intervention is deferred and delivered at six-months. As the practice nurse is the target of the educational intervention, the design appropriately takes the practice as the unit of randomisation. The trial is being managed by the Centre for Primary Health Care Studies, University of Warwick, UK. Ethical approval was granted by the Northern and Yorkshire Multi-centre Research Ethics Committee in June 2004.

### Practices and participants

GP practices from three Strategic Health Authorities in central England have been recruited. Practice eligibility is i) employment of a practice nurse who has undertaken post-registration diabetes care training and ii) use of a laboratory in which HbA1c ranges and assays are DCCT aligned [27] determined and stable. Patient eligibility criteria are adults with type 2 diabetes, not taking insulin and able to read and write English. During the first 12 months of the study, eligible patients had a most recent HbA1c over 8%. We experienced low recruitment over this period as trial commencement concurred with the implementation of a payment linked quality improvement policy [28] during which time GPs have become more aggressive in the prescribing of oral hypoglycemic agents and insulin in an attempt to reduce each patient's HbA1c to below the maximum payment threshold of 7.4%. This has resulted in considerably fewer patients than anticipated meeting the eligibility criteria. It also means that eligible patients include a greater proportion of those for whom more aggressive pharmacological management was not working either due to poor attendance at reviews, complex diabetes management situations or treatment concordance issues. One year into the trial a protocol change was agreed by the investigators, funders and the research ethical committee to enhance patient recruitment by reducing the patient eligibility to a minimum of HbA1c 7%.

### Screening eligibility

**Figure 1:**
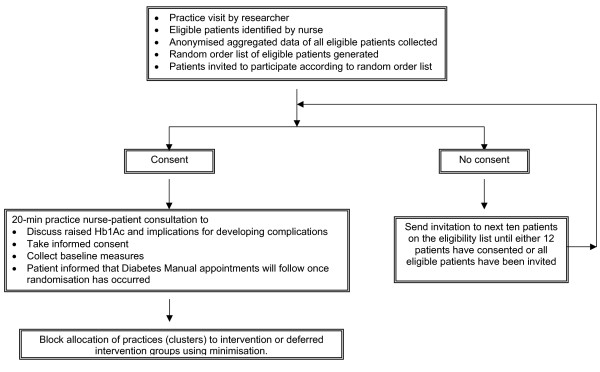
Flowchart of trial from recruitment to randomisation.

Participating practices identified eligible patients from practice registers and a researcher visited to collect aggregated data for all eligible patients and generate a random ordered recruitment list [figure 1]. Aggregated, anonymised HbA1c, blood pressure (BP), body mass index (BMI) and basic demographic data of the eligible practice populations were collected prior to recruitment to allow any difference between the actual study participants and the eligible population to be identified. Patients were invited to participate by the nurse according to the random order list. This ensured that patients were invited consecutively on the grounds of eligibility rather than on the grounds of anticipated concordance by the practice nurse. The list was followed until 12 patients were recruited or the list was exhausted. All patients were recruited prior to practice cluster randomisation.

### Baseline assessment

Baseline clinical assessment was conducted by the practice nurse prior to randomisation during an additional diabetes review. Blood samples for HbA1c assessment, total and HDL cholesterol were taken by the nurse and analysed outside the practice by the DCCT aligned [27] laboratory blinded to practice or patient group allocation. A self-completion booklet of questionnaires consisting of demographic questions, the problem areas in diabetes scale (PAID) [29], the diabetes management self-efficacy scale (DMSES) [30] and the diabetes self-care activities measure [31] was given to the patient by the nurse at baseline for return in replied paid envelope to the research team. Process data consisted of the diabetes self-care activities scale [31] and prescribed medication collected by the practice nurse during clinical assessment. Demographic data includes age, gender, net weekly income, highest educational qualification, ethnicity and duration of diabetes.

### Outcome assessment

Follow up for all measures is at six months post randomisation for both arms and 12 months post randomisation for the intervention arm to assess maintenance of effect on all measures. Six and 12-month clinical assessment is carried out by the practice nurse during a diabetes review. Psychosocial measures are collected by self-completion patient postal questionnaire.

### Group allocation

**Figure 2:**
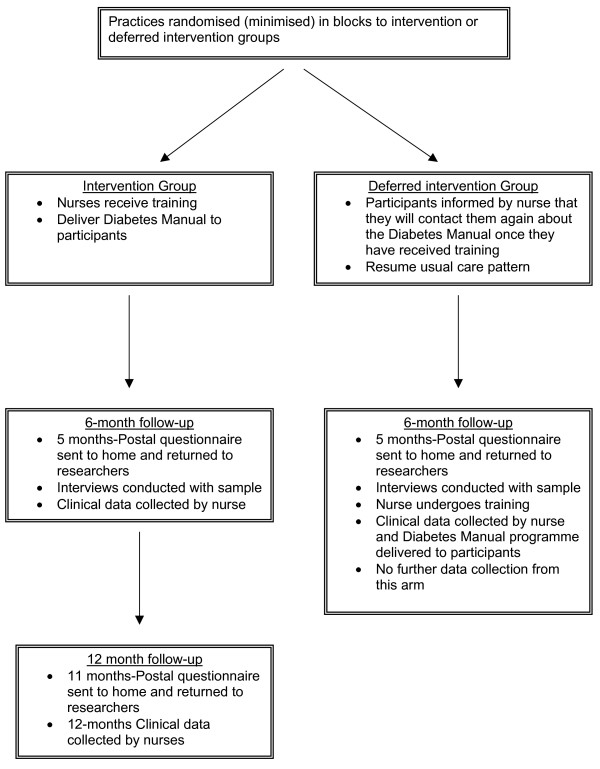
Trial flowchart from randomisation to completion at 12 month follow-up.

Following patient baseline data collection, practices were minimised [32] into intervention and control arms according to mean HbA1c of recruited patients, practice self-assessed quality of diabetes care indicators [28] and the number of individuals recruited [figure 2]. Computer-aided minimisation was undertaken by a statistician blind to practice identity. All members of the research team who will be inputting, analysing and interpreting both qualitative and quantitative data are blind to the group allocation of practices and participants. The nurse trainer, one research fellow and one secretary remain unblinded to ensure effective communication and support between the research team, the practices and the participants regarding training events and follow-up data collection.

### The interventions

The intervention arm practice nurses undertake a two-day training in facilitation skills associated with use of the Diabetes Manual, including practical skill development and underlying theoretical principles of self-efficacy [see Additional file 1]. The nurse training syllabus incorporates efficacy enhancing mechanisms and therefore provides an experiential model for the nurses. Recruited patients have a 20 minute consultation with the trained nurse to introduce the 12-week Diabetes Manual programme. The first two patients that each nurse delivers the intervention to are intended to be practise consultations with ineligible patients to ensure she has gained familiarity with the intervention delivery procedures. The control arm receives usual care for a period of six months. Following six-month data collection, control arm nurses receive the training and deliver the Diabetes Manual to the recruited patients in their practice. No further trial data is collected from control arm participants thereafter.

### Trial management

A trial steering group has been formed consisting of the full research team, the nurse trainer, a practice nurse and two lay people from the Warwick Diabetes Care Research User Group. Due to the low risk nature of the interventions, a data monitoring committee is not necessary.

### Quality assurance

Standardised delivery of the interventions will be assured by i) the nurse training programme being accompanied by detailed written material to which the nurses can refer during the intervention delivery; ii) analysis of a telephone support data sheet used by the nurses to prompt and record telephone discussion; iii) the nurse audio records 1 in 6 telephone support calls and the teaching and research team listen to half of these calls, and assesses them against the training protocol for telephone support and iv) 12 patients in each arm will be invited to participate in a semi-structured interview, and this data will be analysed to provide evidence of protocol adherence by the nurses for a selection of the patients and programme adherence by the patients.

### Qualitative Study

#### Patient interviews

Twelve semi-structured interviews are being undertaken with a purposive sample from each arm of the trial (n = 24). Sampling aims to capture differing self-efficacy levels and educational attainment and practice location. The interviews will explore any differences in the intervention and control arm experience of diabetes management during the trial period. The interviews are completed during a two-week period following completion of the six-month questionnaire data and before the collection of follow-up clinical data so that the interview does not influence the questionnaire completion. The interviews' close proximity to the clinical follow-up review will limit its influence on clinical outcomes such as HbA1c.

#### Health professionals' views

Two focus groups will be held following six-month data collection for trained, intervention arm, practice nurses to capture their perspective of the Diabetes Manual programme.

### Sample size and rate of recruitment

We originally sought power of 90% (alpha = 0.05) to detect a 0.6% difference in individual patient HbA1c between intervention and control arms. Based on feasibility work, we estimated that the number of patients who have type 2 diabetes per recruited practice that meet the inclusion criteria would be an average of 80 so it would be reasonable for each practice to recruit and manage between 5–12 patients (expected mean number of patients = 10). Using an intra cluster correlation for HbA1c of 0.043 and allowing for 15% patient attrition and variability in clusters size gives an adjusted sample size of 212 in each arm. Allowing for an attrition rate of 10% for practices, we aimed to recruit a total of 46 practices, each willing to recruit up to12 patients. Because of the lower rate of patient recruitment in the first twelve months, we expected fewer patients per practice. We compensated for this by recruiting more practices. We estimated that with analysable data on five patients per practice in 50 practices the power of our study to detect a 0.6% difference would be 80% (accounting for between cluster correlation and variable cluster size as described above). This gives a lower power than we originally aimed for but was a realistic target.

### Statistical analysis

Data is double entered and discrepancy checks carried out with source data. To account for clustering by practice, primary and secondary outcomes will be analysed using population averaged models with robust standard errors (using generalised estimating equations). Patient and practice level covariates thought to be related to outcome, including practice self-assessed quality of diabetes care indicators [28], geographical location of practice, level of patient outcome at baseline (where available), and patient age, gender, education and socio-economic status will be considered for incorporation in the analysis. Covariates will be included if they show a strong (α<10%) relationship with outcome. We will also include a covariate representing whether the practice was recruited before or after the change in protocol relating to HbA1c levels. To detect the presence of any significant differences in effect between sub-groups, we will fit first order interaction terms for interactions between intervention and the following factors which represent the sub-groups of interest: laboratory used for blood sample analysis, patient age and educational attainment. Separate models will also be fitted for the separate subgroups, but these results will be exploratory rather than definitive. The questions we aim to explore in the subgroup analyses are: Is the effect on HbA1c different for patients recruited before and after the protocol change or for patients in different age groups? Is any effect on self-efficacy different for older and younger patients or by educational attainment?

## Discussion

This trial aims to evaluate the efficacy of an intervention that offers structured diabetes education and theoretically informed self-management skills training. This delivery of both educational and psychological components in a single programme offers the potential for people to learn about their diabetes in a psychologically motivating and confidence enhancing structure. The systematic reviews of both Hampson et al [12] and Ismail et al [10] indicate the need to develop and evaluate these combined approaches to ensure that treatment concordance between patients and health professionals can be achieved. Within our intervention the patient is encouraged how to use physical activity, dietary intake and oral medication to understand and regulate their blood glucose levels. In the absence of insulin treatment, these are the principle mechanisms by which glucose control can be maintained on an hour by hour basis for people with type 2 diabetes. This will further test the biomedical feedback hypothesis identified as important in the Gibson et al [13] and Norris et al [15] systematic reviews of long-term condition management. This trial uses self-efficacy theory throughout to train the nurses, to promote diabetes related changes in behaviour and as an outcome measure consisting of specific diabetes self-management activities. Consistency of theoretical approach will increase our understanding of the diabetes related health behaviours that are more or less susceptible to change through efficacy enhancing mechanisms and how this impacts on clinical outcomes. Data relating to intervention fidelity on a theoretical level by the nurse facilitators will offer valuable understanding about the relative importance of theoretically-based nurse-patient interaction on the clinical and psychosocial outcomes experienced by patients.

The Ismail et al meta-analysis [10] included a number of trials where psychological interventions were delivered by generalist health professionals (non-mental health trained) but did not focus on the content of the training delivered to the nurses to enable clinically significant reductions in HbA1c to be achieved. The current trial offers psychological training to generalist professionals (practice nurses) and the quality assurance procedures will enable clinical outcome data to be compared to quality assurance data where psychological intervention fidelity by the nurses was both strong and weak. This will provide evidence on which to base further training syllabus and programme development for psychological interventions.

This complex intervention, consisting of five distinct components, has undergone development informed by the UK Medical Research Council Framework for Development and Evaluation of Complex Interventions [33]. Underlying theoretical principles have been developed and tested and the forerunning Heart Manual provided phase I data to inform the Diabetes Manual development. The Diabetes Manual has been developed in identical component format to the Heart Manual and demonstrated strong face validity with both health professionals and people with type 2 diabetes. Due to the weight of Heart Manual evidence [22-24]. Intervention delivery quality assurance procedures and patient interview data will enable us to assess this transferability. With the growth of generic chronic disease interventions internationally [17, 34, 35], the trial findings will contribute to the debate regarding the efficacy of such approaches.

The protocol change regarding patient HbA1c eligibility represents a trial limitation, and presenting the consort diagram and the data interpretation will be more challenging as a result.

## Competing interests

The author(s) declare that they have no competing interests.

## Authors' contributions

JS and HH conceived the development of the intervention. JS, HH and AF developed the intervention. All authors developed the trial protocol and contributed to drafting the manuscript. SE resolved statistical issues. JS, JD, AF and HH have managed the running of the trial.

## Investigators and trial steering group

Members of the writing group for this manuscript were JS, AF, JD, HH and SE.

The investigators are: J Sturt, A Farmer, H Hearnshaw, J Dale, F Griffiths, S Eldridge, J Barlow and P O'Hare.

The steering group consists of the investigators and C Fox, S Whitlock, A Lindenmeyer, M Wakelin, R Walker, D Karet, M Kingdom and G Freeman.

## Funding

The trial is funded by a Diabetes UK structured education project grant and NHS R&D Support funding. JS is funded by a Department of Health NCCRCD post-doctoral award.

## Pre-publication history

The pre-publication history for this paper can be accessed here:



## Supplementary Material

Additional File 1 Diabetes Manual programme and training syllabus. Diabetes Manual programme components and the accompanying nurse training syllabus to prepare for effective delivery of the intervention to patients. Click here for file
