# Supplementary material for: The Diabetes Manual trial protocol – a cluster randomized controlled trial of a self-management intervention for type 2 diabetes [ISRCTN06315411]
Source: BMC Fam Pract. 2006 Jul 17;7:45. doi: 10.1186/1471-2296-7-45 (PMC1555586; doi:10.1186/1471-2296-7-45)
Supplement: Additional File 1 — Diabetes Manual programme and training syllabus. Diabetes Manual programme components and the accompanying nurse training syllabus to prepare for effective delivery of the intervention to patients. [file 1471-2296-7-45-S1.doc]

| **Component** | **Programme content** | **Training syllabus** |
| --- | --- | --- |
| 1 | 2-day training for practice nurses (PN) experienced in diabetes management. | Self-efficacy theory and adult learning.  Intervention structure  Practical skill development in telephone support and empowering communication |
| 2 | Patient manual completed over 12 weeks | Diabetes facts / Metabolism / Goal setting and evaluation / Exercise/ Nutrition / Blood glucose monitoring / Weight loss / Smoking cessation / tests/ Complications / Medication / Vignettes / Stress, anxiety and depression/ Cholesterol. |
| 3 | Relaxation audiotape | Teach techniques and facilitate opportunities |
| 4 | Frequently asked questions audiotape | Provides for quick diabetes self-management recall for patient and carers/family |
| 5 | Telephone support from practice nurse in weeks 1,5 and 11. | Assess goal progress; patient recollection of goal achievement, promotion of goal self- evaluation and re-negotiation. |
